# 5,5-Dimethyl-2-methyl­seleno-1,3,2-dioxaphospho­rinan-2-one

**DOI:** 10.1107/S1600536810008330

**Published:** 2010-03-17

**Authors:** Grzegorz Cholewinski, Jaroslaw Chojnacki, Jerzy Pikies, Janusz Rachon

**Affiliations:** a Chemical Faculty, Gdansk University of Technology, Narutowicza 11/12, Gdansk PL-80233, Poland

## Abstract

The title compound, C_6_H_13_O_3_PSe, was obtained in the reaction of 5,5-dimethyl-2-oxo-2-seleno-1,3,2-dioxaphospho­r­inane potassium salt with methyl iodide. The seleno­methyl group is in the axial position in relation to the six-membered dioxaphospho­rinane ring.

## Related literature

For the structures of similar methyl esters with >P(Se)OMe and >P(Se)SeMe groups, see: Grand *et al.* (1975 ▶); Bartczak *et al.* (1987 ▶). For 5,5-dimethyl-2-seleno-1,3,2-dioxaphospho­rin­ane derivatives with equatorial Se atoms, see: Bartczak & Wolf (1983 ▶); Bartczak *et al.* (1983 ▶); Wolf & Bartczak (1989 ▶) and for *O*-acyl derivatives with equatorial selenium, see: Cholewinski *et al.* (2009 ▶). For conformers with axial Se atoms, see: Bartczak *et al.* (1986 ▶); Potrzebowski *et al.* (1994 ▶); Wieczorek *et al.* (1995 ▶). For details of the synthesis, see: Rachon *et al.* (2005 ▶); Stec (1974 ▶). For a description of the Cambridge Structural Database, see: Allen (2002 ▶).
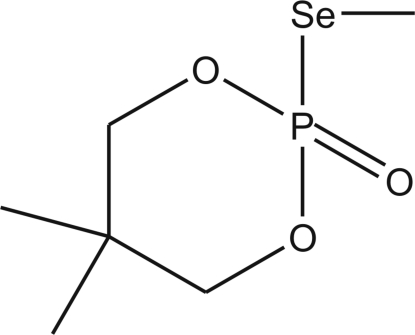


## Experimental

### 

#### Crystal data

C_6_H_13_O_3_PSe
*M* *_r_* = 243.09
Monoclinic, 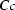

*a* = 9.2252 (4) Å
*b* = 9.4842 (4) Å
*c* = 11.4160 (6) Å
β = 101.078 (5)°
*V* = 980.22 (8) Å^3^
*Z* = 4
Mo *K*α radiation
μ = 3.96 mm^−1^
*T* = 150 K
0.59 × 0.41 × 0.28 mm

#### Data collection

Oxford Diffraction KM-4-CCD diffractometer
Absorption correction: analytical [*CrysAlis RED* (Oxford Diffraction, 2009 ▶), using a multifaceted crystal model based on expressions derived by Clark & Reid (1995 ▶)] *T*_min_ = 0.179, *T*_max_ = 0.372
3146 measured reflections
1238 independent reflections
1214 reflections with *I* > 2σ(*I*)
*R*_int_ = 0.045

#### Refinement

*R*[*F* ^2^ > 2σ(*F* ^2^)] = 0.026
*wR*(*F* ^2^) = 0.065
*S* = 1.05
1238 reflections
103 parameters
2 restraints
H-atom parameters constrained
Δρ_max_ = 0.69 e Å^−3^
Δρ_min_ = −0.33 e Å^−3^
Absolute structure: Flack (1983 ▶), 189 Friedel pairs
Flack parameter: −0.009 (10)

### 

Data collection: *CrysAlis CCD* (Oxford Diffraction, 2009 ▶); cell refinement: *CrysAlis RED* (Oxford Diffraction, 2009 ▶); data reduction: *CrysAlis RED*; program(s) used to solve structure: *SHELXS97* (Sheldrick, 2008 ▶); program(s) used to refine structure: *SHELXL97* (Sheldrick, 2008 ▶); molecular graphics: *ORTEP-3 for Windows* (Farrugia, 1997 ▶) and *Mercury* (Macrae *et al.*, 2006 ▶); software used to prepare material for publication: *WinGX* (Farrugia, 1999 ▶), *PLATON* (Spek, 2009 ▶) and *publCIF* (Westrip, 2010 ▶).

## Supplementary Material

Crystal structure: contains datablocks global, I. DOI: 10.1107/S1600536810008330/dn2544sup1.cif

Structure factors: contains datablocks I. DOI: 10.1107/S1600536810008330/dn2544Isup2.hkl

Additional supplementary materials:  crystallographic information; 3D view; checkCIF report

## Figures and Tables

**Fig. 1.**
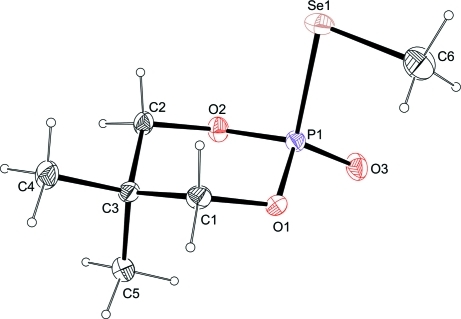
The nolecular structure of (I), with the atom labeling scheme. Displacement ellipsods are drawn at the 30% probability level. H atoms are represented as small spheres of arbitrary radii.

## supplementary crystallographic information

### Comment

The title compound, 5,5-dimethyl-2-methylseleno-2-oxo-1,3,2-dioxaphosphorinane, forms molecular crystals (Fig. 1). No stronger intermolecular interactions beside weak C–H···O=P contacts (the shortest H6c···O3 distance is 2.387 Å) can be found. Bonds P–Se and Se–C in the selenomethyl group are almost perpendicular, which is expected for selenium compounds. For comparison: in related compound bearing >P(Se)SeMe moiety (Bartczak *et al.*, 1987) the relevant angle is ca two degrees wider (95.17°). Rather long P–Se bond length of ca 2.2 Å is typical for selenium with the coordination number two.

Selenium atom can adopt axial or equatorial positions in the chair conformation of the six-membered ring in derivatives of 5,5-dimethyl-2-seleno-1,3,2-dioxaphosphorinane. Search of CSD data (Allen, 2002) reveals both possibilities can be realised in the solid state structures. Derivatives, which are substituted at P atom by –NH–aryl group, often have equatorial Se atoms (Bartczak *et al.*, 1983, Bartczak & Wolf, 1983, Wolf & Bartczak, 1989 and Grand *et al.*, 1975). Recently, we reported on several *O*-acyl derivatives with equatorial Se, but also –NH_2_ and NH–C(O)^t^Bu derivatives, which contain selenium atom in axial positions (Cholewinski *et al.*, 2009). More precisely, the last derivative contains both conformers - axial and equatorial - in the unit cell. Conformers with axial Se atoms were found also for –NHEt derivative (Bartczak *et al.*, 1986), and for two compounds with double P=O or P=S bonds: the bisselenide and the bisdiselenide, respectively (Wieczorek *et al.*, 1995 and Potrzebowski *et al.*, 1994). In the case of 5,5-dimethyl-2-methylseleno-1,3,2-dioxaphosphorinane-2-selenide the group –SeMe is aligned in the axial position and P=Se positioned equatorially (Bartczak *et al.*, 1987). In 5,5-dimethyl-2-methoxy-2-seleno-1,3,2-dioxaphosphorinane –OMe is axial, so Se atom adopts the equatorial position (Grand *et al.*, 1975).

In our previous study (Cholewinski *et al.*, 2009) we described a correlation between the anomeric iteractions *n_O_*→σ*_P–X_ (where X is O or NH) and axial / equatorial conformer distribution in >P(Se)XR systems. However, those orbital systems were different - contained single P–X bond and the selenium atom was linked only to P atom, formally by a double bond. The reasoning derived there cannot be applied to prediction of conformation for systems with double P=O and single P–Se bonds, like the present case or to bisselenides. In fact, the doubly bonded oxygen atoms tend to occupy equatorial position in relation to the six-membered ring.

### Experimental

The title compound was obtained according to Stec, 1974. To a solution of 5,5-dimethyl-2-oxo-2-seleno-1,3,2-dioxaphosphorinane potassium salt (Rachon *et al.*, 2005) (1 mmol) in THF (5 ml) was added methyl iodide (1 mmol) portionwise. The reaction mixture was stirred at room temperature for 15 min. Then, the solvent was evaporated and crude product crystallized from hexane. Re-crystallization from CH_2_Cl_2_ – petroleum ether (bp 40 – 60 °C) gave product in 53% yield.

Mp 90.5-92 °C, ^31^P NMR (THF + C_6_D_6_) δ = 11.5 ppm, ^1^J_PSe_ =456 Hz, IR ν(cm^-1^): P=O 1258.

Literature data (Stec, 1974): mp 90.5-91.5 °C; ^31^P NMR (methanol) δ = 13.1 ppm, ^1^J_PSe _= 457 Hz.

### Refinement

Hydrogen atoms were placed in calculated positions and refined using a standard riding model. C–H bond lengths were set to 0.99 and 0.98 Å and *U_iso_*(H) were set to 1.5 and 1.2 *U_eq_*(C) for CH_3_ and CH_2_ groups, respectively.

The residual electron density peak is 0.83 Å from SE1, the deepest electron density hole is 1.28 Å from H5A. Absolute structure determination is unequivocal because only 189 Bijvoet pairs were measured. As the structure is not chiral, we did not attempt to elucidate it further.

### Crystal data

**Table:**

C_6_H_13_O_3_PSe	*F*(000) = 488
*M**_r_* = 243.09	*D*_x_ = 1.647 Mg m^−^^3^
Monoclinic, *C**c*	Melting point: 364(1) K
Hall symbol: C -2yc	Mo *K*α radiation, λ = 0.71073 Å
*a* = 9.2252 (4) Å	Cell parameters from 3018 reflections
*b* = 9.4842 (4) Å	θ = 3.1–28.6°
*c* = 11.4160 (6) Å	µ = 3.96 mm^−^^1^
β = 101.078 (5)°	*T* = 150 K
*V* = 980.22 (8) Å^3^	Needless, colourless
*Z* = 4	0.59 × 0.41 × 0.28 mm

### Data collection

**Table:**

Oxford Diffraction KM-4-CCD diffractometer	1238 independent reflections
Radiation source: Mo Ka radiation	1214 reflections with *I* > 2σ(*I*)
graphite	*R*_int_ = 0.045
Detector resolution: 8.1883 pixels mm^-1^	θ_max_ = 27°, θ_min_ = 3.1°
ω scans, 0.8° width	*h* = −11→11
Absorption correction: analytical [*CrysAlis RED* (Oxford Diffraction, 2009), using a multifaceted crystal model based on expressions derived by Clark & Reid (1995)]	*k* = −11→11
*T*_min_ = 0.179, *T*_max_ = 0.372	*l* = −5→14
3146 measured reflections	

### Refinement

**Table:**

Refinement on *F*^2^	Secondary atom site location: difference Fourier map
Least-squares matrix: full	Hydrogen site location: inferred from neighbouring sites
*R*[*F*^2^ > 2σ(*F*^2^)] = 0.026	H-atom parameters constrained
*wR*(*F*^2^) = 0.065	*w* = 1/[σ^2^(*F*_o_^2^) + (0.0472*P*)^2^] where *P* = (*F*_o_^2^ + 2*F*_c_^2^)/3
*S* = 1.05	(Δ/σ)_max_ = 0.005
1238 reflections	Δρ_max_ = 0.69 e Å^−^^3^
103 parameters	Δρ_min_ = −0.33 e Å^−^^3^
2 restraints	Absolute structure: Flack (1983), 189 Friedel pairs
Primary atom site location: structure-invariant direct methods	Flack parameter: −0.009 (10)

### Special details

**Table:**

Experimental. CrysAlis RED (Oxford Diffraction, 2009), Analytical numeric absorption correction using a multifaceted crystal model based on expressions derived by Clark & Reid (1995).
Geometry. All s.u.'s (except the s.u. in the dihedral angle between two l.s. planes) are estimated using the full covariance matrix. The cell s.u.'s are taken into account individually in the estimation of s.u.'s in distances, angles and torsion angles; correlations between s.u.'s in cell parameters are only used when they are defined by crystal symmetry. An approximate (isotropic) treatment of cell s.u.'s is used for estimating s.u.'s involving l.s. planes.
Refinement. Refinement of *F*^2^ against ALL reflections. The weighted *R*-factor wR and goodness of fit *S* are based on *F*^2^, conventional *R*-factors *R* are based on *F*, with *F* set to zero for negative *F*^2^. The threshold expression of *F*^2^ > 2σ(*F*^2^) is used only for calculating *R*-factors(gt) etc. and is not relevant to the choice of reflections for refinement. *R*-factors based on *F*^2^ are statistically about twice as large as those based on *F*, and *R*- factors based on ALL data will be even larger.

### Fractional atomic coordinates and isotropic or equivalent isotropic displacement parameters (Å^2^)

**Table:**

	*x*	*y*	*z*	*U*_iso_*/*U*_eq_	
Se1	0.50195 (3)	0.96219 (3)	0.92664 (3)	0.03344 (13)	
P1	0.69226 (10)	0.84725 (9)	0.88178 (8)	0.02227 (18)	
O1	0.7985 (3)	0.9613 (2)	0.8432 (2)	0.0256 (6)	
O2	0.7776 (3)	0.7862 (3)	1.0038 (2)	0.0268 (5)	
O3	0.6521 (3)	0.7389 (3)	0.7910 (3)	0.0352 (6)	
C1	0.8748 (4)	1.0581 (4)	0.9339 (3)	0.0264 (7)	
H1A	0.8017	1.1201	0.9614	0.032*	
H1B	0.9427	1.1185	0.8986	0.032*	
C2	0.8592 (4)	0.8836 (4)	1.0927 (3)	0.0267 (7)	
H2A	0.9173	0.8285	1.1592	0.032*	
H2B	0.7882	0.9426	1.1257	0.032*	
C3	0.9621 (4)	0.9780 (4)	1.0400 (3)	0.0234 (7)	
C4	1.0265 (5)	1.0859 (5)	1.1365 (4)	0.0352 (8)	
H4A	0.9463	1.1423	1.1576	0.053*	
H4B	1.0957	1.1479	1.1061	0.053*	
H4C	1.0785	1.0361	1.2075	0.053*	
C5	1.0866 (4)	0.8934 (4)	1.0014 (4)	0.0317 (8)	
H5A	1.1587	0.9584	0.9783	0.048*	
H5B	1.0453	0.8332	0.9334	0.048*	
H5C	1.1353	0.8345	1.0679	0.048*	
C6	0.4408 (6)	1.0359 (5)	0.7641 (5)	0.0502 (13)	
H6A	0.4241	0.9573	0.7075	0.075*	
H6B	0.5184	1.0973	0.7449	0.075*	
H6C	0.3493	1.0901	0.7588	0.075*	

### Atomic displacement parameters (Å^2^)

**Table:**

	*U*^11^	*U*^22^	*U*^33^	*U*^12^	*U*^13^	*U*^23^
Se1	0.02636 (18)	0.0350 (2)	0.0420 (2)	0.00387 (16)	0.01415 (14)	−0.0031 (2)
P1	0.0210 (4)	0.0227 (4)	0.0225 (4)	0.0011 (3)	0.0029 (3)	−0.0025 (4)
O1	0.0235 (13)	0.0358 (15)	0.0182 (11)	−0.0015 (9)	0.0056 (10)	0.0014 (10)
O2	0.0287 (12)	0.0229 (11)	0.0273 (12)	−0.0058 (9)	0.0014 (10)	0.0014 (11)
O3	0.0294 (13)	0.0375 (13)	0.0351 (14)	0.0044 (12)	−0.0027 (11)	−0.0129 (13)
C1	0.0289 (18)	0.0251 (15)	0.0266 (17)	−0.0075 (14)	0.0088 (15)	0.0013 (15)
C2	0.0282 (16)	0.0323 (17)	0.0189 (14)	−0.0088 (13)	0.0029 (13)	0.0021 (14)
C3	0.0240 (17)	0.0262 (17)	0.0210 (15)	−0.0061 (13)	0.0065 (14)	−0.0029 (14)
C4	0.039 (2)	0.0370 (19)	0.0292 (18)	−0.0188 (17)	0.0060 (15)	−0.0077 (18)
C5	0.0253 (18)	0.039 (2)	0.0296 (18)	0.0000 (14)	0.0021 (14)	−0.0003 (18)
C6	0.044 (3)	0.054 (3)	0.050 (3)	0.026 (2)	0.003 (2)	0.005 (2)

### Geometric parameters (Å, °)

**Table:**

Se1—C6	1.962 (6)	C2—H2B	0.99
Se1—P1	2.2094 (9)	C3—C5	1.534 (5)
P1—O3	1.456 (3)	C3—C4	1.537 (5)
P1—O2	1.574 (3)	C4—H4A	0.98
P1—O1	1.579 (3)	C4—H4B	0.98
O1—C1	1.460 (4)	C4—H4C	0.98
O2—C2	1.468 (4)	C5—H5A	0.98
C1—C3	1.523 (5)	C5—H5B	0.98
C1—H1A	0.99	C5—H5C	0.98
C1—H1B	0.99	C6—H6A	0.98
C2—C3	1.512 (5)	C6—H6B	0.98
C2—H2A	0.99	C6—H6C	0.98
			
C6—Se1—P1	93.09 (15)	C1—C3—C5	110.1 (3)
O3—P1—O2	112.74 (15)	C2—C3—C4	107.1 (3)
O3—P1—O1	111.84 (16)	C1—C3—C4	108.2 (3)
O2—P1—O1	105.49 (14)	C5—C3—C4	110.3 (3)
O3—P1—Se1	114.08 (12)	C3—C4—H4A	109.5
O2—P1—Se1	105.16 (11)	C3—C4—H4B	109.5
O1—P1—Se1	106.89 (10)	H4A—C4—H4B	109.5
C1—O1—P1	118.3 (2)	C3—C4—H4C	109.5
C2—O2—P1	119.0 (2)	H4A—C4—H4C	109.5
O1—C1—C3	111.1 (3)	H4B—C4—H4C	109.5
O1—C1—H1A	109.4	C3—C5—H5A	109.5
C3—C1—H1A	109.4	C3—C5—H5B	109.5
O1—C1—H1B	109.4	H5A—C5—H5B	109.5
C3—C1—H1B	109.4	C3—C5—H5C	109.5
H1A—C1—H1B	108	H5A—C5—H5C	109.5
O2—C2—C3	112.1 (3)	H5B—C5—H5C	109.5
O2—C2—H2A	109.2	Se1—C6—H6A	109.5
C3—C2—H2A	109.2	Se1—C6—H6B	109.5
O2—C2—H2B	109.2	H6A—C6—H6B	109.5
C3—C2—H2B	109.2	Se1—C6—H6C	109.5
H2A—C2—H2B	107.9	H6A—C6—H6C	109.5
C2—C3—C1	109.6 (3)	H6B—C6—H6C	109.5
C2—C3—C5	111.5 (3)		
			
C6—Se1—P1—O3	−61.8 (2)	P1—O1—C1—C3	54.9 (4)
C6—Se1—P1—O2	174.2 (2)	P1—O2—C2—C3	−51.4 (4)
C6—Se1—P1—O1	62.4 (2)	O2—C2—C3—C1	56.3 (4)
O3—P1—O1—C1	−166.5 (2)	O2—C2—C3—C5	−65.9 (4)
O2—P1—O1—C1	−43.6 (3)	O2—C2—C3—C4	173.4 (3)
Se1—P1—O1—C1	68.0 (3)	O1—C1—C3—C2	−58.0 (4)
O3—P1—O2—C2	163.9 (3)	O1—C1—C3—C5	65.0 (4)
O1—P1—O2—C2	41.6 (3)	O1—C1—C3—C4	−174.5 (3)
Se1—P1—O2—C2	−71.2 (3)		
